# Correction To: Intranasal booster using an Omicron vaccine confers broad mucosal and systemic immunity against SARS-CoV-2 variants

**DOI:** 10.1038/s41392-023-01448-x

**Published:** 2023-05-24

**Authors:** Qian Wang, Chenchen Yang, Li Yin, Jing Sun, Wei Wang, Hengchun Li, Zhengyuan Zhang, Si Chen, Bo Liu, Zijian Liu, Linjing Shi, Xiaolin Liu, Suhua Guan, Chunhua Wang, Linbing Qu, Ying Feng, Xuefeng Niu, Liqiang Feng, Jincun Zhao, Pingchao Li, Ling Chen, Nanshan Zhong

**Affiliations:** 1 State Key Laboratory of Respiratory Disease, Guangzhou Institute of Respiratory Health, The First Affiliated Hospital of Guangzhou Medical University, Guangzhou, China; 2 Guangzhou nBiomed Ltd., Guangzhou, China; 3 Guangzhou Laboratory & Bioland Laboratory, Guangzhou, China; 4 Guangdong Laboratory of Computational Biomedicine, Guangzhou Institutes of Biomedicine and Health, Chinese Academy of Sciences, Guangzhou, China; 5 University of Chinese Academy of Sciences, Beijing, China

**Keywords:** Vaccines, Adaptive immunity

Correction to: *Signal Transduction and Targeted Therapy* 10.1038/s41392-023-01423-6, published online 17 April 2023

After online publication of the article^1^, the authors noticed two inadvertent mistakes occurred in References that needs to be corrected. The correct text is provided as follows.

In the version of this article initially published, there are two errors in References 11 and 15. The correct Reference 11 is “https://www.who.int/publications/m/item/global-covid-19-vaccination-strategy-in-a-changing-world--july-2022-update”. The correct Reference 15 is “Van Kampen, K. R. et al. Safety and immunogenicity of adenovirus-vectored nasal and epicutaneous influenza vaccines in humans. *Vaccine*. **23**, 1029–1036 (2005)”. The rest of the References remain the same, and the interpretation of the results remains unchanged.

The original article has been corrected
